# ^68^Ga-Galmydar: A PET imaging tracer for noninvasive detection of Doxorubicin-induced cardiotoxicity

**DOI:** 10.1371/journal.pone.0215579

**Published:** 2019-05-23

**Authors:** Jothilingam Sivapackiam, Shivesh Kabra, Sylvia Speidel, Monica Sharma, Richard Laforest, Amber Salter, Michael P. Rettig, Vijay Sharma

**Affiliations:** 1 Mallinckrodt Institute of Radiology, Washington University School of Medicine, St. Louis, MO, United States of America; 2 Pediatrics Cardiology, Washington University School of Medicine, St. Louis, MO, United States of America; 3 Department of Biostatistics, Washington University School of Medicine, St. Louis, MO, United States of America; 4 Division of Oncology, Department of Medicine, Washington University School of Medicine, St. Louis, MO, United States of America; 5 Department of Neurology, Washington University School of Medicine, St. Louis, MO, United States of America; 6 Department of Biomedical Engineering, School of Engineering & Applied Science, Washington University, St. Louis, MO, United States of America; Wayne State University, UNITED STATES

## Abstract

**Background:**

Cancer patients undergoing Doxorubicin (DOX) treatment are susceptible to acute and chronic cardiac anomalies, including aberrant arrhythmias, ventricular dysfunction, and heart failure. To stratify patients at high risk for DOX -related heart failure (CHF), diagnostic techniques have been sought. While echocardiography is used for monitoring LVEF and LV volumes due to its wide-availability and cost-efficiency, it may not identify early stages of the initiation of DOX-induced systolic heart failure. To address these limitations, PET tracers could also provide noninvasive assessment of early and reversible metabolic changes of the myocardium.

**Objective:**

Herein, we report a preliminary investigation of ^68^Ga-Galmydar potential to monitor Dox-induced cardiomyopathy *in vivo*, *ex vivo*, and *in cellulo* employing both nuclear- and optical imaging.

**Methods and results:**

To assess ^68^Ga-Galmydar ability for monitoring DOX-induced cardiomyopathy, microPET imaging was performed 5 d post treatment of rats either with a single dose of DOX (15 mg/kg) or vehicle as a control (saline) and images were co-registered for anatomical reference using CT. Following tail-vein injection of the radiotracer in rats at 60 min, micro-PET/CT static scan (10 min acquisition), ^68^Ga-Galmydar demonstrated 1.91-fold lower uptake in hearts of DOX-treated (standard uptake value; SUV: 0.92, n = 3) rats compared with their vehicle treated (SUV: 1.76, n = 3) control counterparts. For correlation of PET imaging data, post-imaging quantitative biodistribution studies were also performed, wherein excised organs were counted for γ activity, and normalized to injected dose. The post imaging pharmacokinetic data also demonstrated heart uptake values of 2.0 fold lower for DOX treated rats(%ID/g; DOX: 0.44 ± 0.1, n = 3) compared to their vehicle-treated controls (%ID/g; Control: 0.89 ± 0.03, n = 3, p = 0.04). Employing the fluorescent traits of Galmydar, live cell fluorescence imaging indicated a gradual decrease in uptake and retention of Galmydar within mitochondria of H9c2 cells following DOX-treatment, while indicating dose-dependent and time-dependent uptake profiles. Following depolarization of electronegative transmembrane gradients at the mitochondrial membrane, the uptake of the probe was decreased in H9c2 cells, and the uptake profiles were found to be identical, using both fluorescence and radiotracer bioassays. Finally, the decreased uptake of the metalloprobe in H9c2 cells also correlated with caspase-3 expression resulting from DOX-induced cardiotoxicity and cell death.

**Conclusions:**

^68^Ga-Galmydar could provide a noninvasive assessment of DOX-related and likely reversible metabolic changes at earliest stages. Further studies with other chemotherapeutics (potentially capable of inducing cardiomyopathy) are underway.

## Introduction

**Fig 1:**
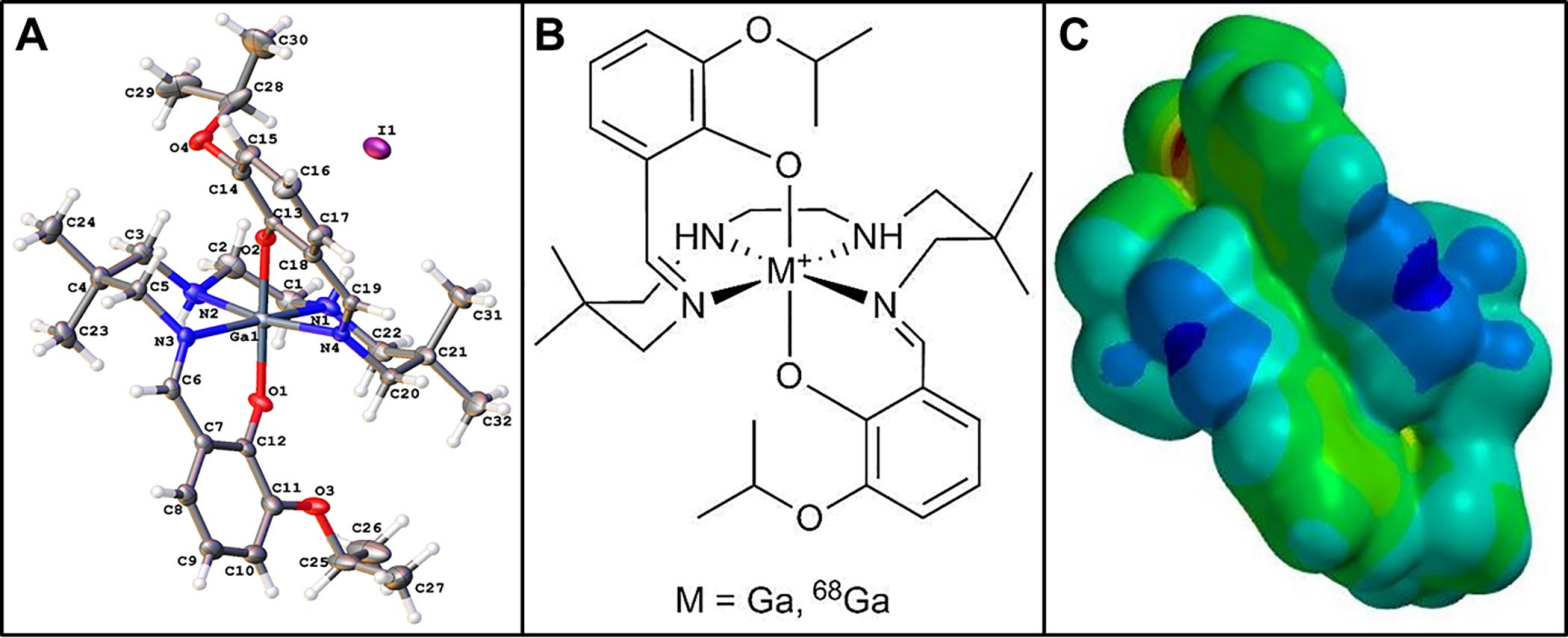
(A) Crystal structure of Galmydar. (B) Chemical Structure. (C) Space Filling Model showing distribution of charge on the molecular surface.

Doxorubicin (DOX; Adriamycin), an anthracycline analogue is a highly versatile chemotherapeutic drug, widely deployed in medical oncology for treating patients with broad spectrum of cancers[1–13], such as acute lymphoblastic leukemia (ALL), acute myeloblastic leukemia (AML), multiple myeloma, neuroblastoma, breast cancer, ovarian cancer, small cell lung cancer, thyroid cancer, head and neck cancer, Hodgkin lymphoma, bone sarcoma, liver, and kidney cancer. Among its mechanism of action, DOX has been postulated to induce its antimitotic and anti-tumor activity by intercalating between DNA base pairs, inhibiting DNA transcription, concomitantly halting protein synthesis, and triggering over-production of free-radical reactive oxygen species (ROS) thus inducing cytotoxic effects indiscriminately including cardiotoxicity [14]. Therefore, the clinical utility of DOX, and other chemotherapeutics in this class of drugs is somewhat curtailed by their putative dose-dependent cardiotoxicity, resulting from the sensitivity of myocardium cells to oxidative stress[15]. The irreversible side-effects of DOX treatment in patients also include dilated cardiomyopathy [15]. These effects are further exacerbated by the fact that the myocardium tissue lacks expression of the adenosine binding cassette (ABC) family of drug transporters, such as Pgp and BCRP [16], which are poised to excrete recognized substrates from targeted tissues thus diminishing cytotoxic effects [15]. Noticeably, symptoms of DOX-induced cardiomyopathy can manifest in patients during or after DOX treatment, pointing to the exigency for a diagnostic probe to discern cardiac alterations in the subclinical stages, in order to mitigate permanent damage[17]. Current diagnostic techniques, such as multigated acquisition scan (MUGA) and echocardiography provide information about DOX related decrease in left ventricular function that may already reflect irreversible cardiac damage [17]. Therefore, mechanistic pathways mediating DOX-induced cardiomyopathy have been intensely debated, including a role of DOX metabolism in the mitochondria [18, 19]. Earlier, we have reported that ^67/68^Ga-Galmydar, could be a useful myocardial perfusion molecular imaging (MPI) agent [20]. Importantly, Galmydar is a cationic, metalloprobe having a fairly uniform distribution of a delocalized positive charge on its molecular surface [21] with overall octahedral geometry (**Fig 1; See Analytical Characterization: S1–S3 Figs, S1 and S2 Tables**). Literature precedents also indicate that mitochondrial dysfunction could be “an early” or “the earliest” indicator of DOX-induced cardiomyopathy [15], wherein downstream activation of effector caspases mediates cell apoptosis [22]. Therefore, molecular imaging agents capable of offering noninvasive and specific detection of mitochondrial function in vivo have been sought. To accomplish this objective, single photon emission computed tomography (SPECT) imaging probes, such as ^99m^Tc-Sestamibi (MIBI: methoxy-isobutylisonitrile) has been investigated for detection of DOX-induced cardiomyopathy. However, the data from ^99m^Tc-Sestamibi studies are conflicting. While the cell assays [23] and human studies [24] showed decreased cardiac-uptake of ^99m^Tc-Sestamibi upon DOX treatment, animal studies [25] conversely revealed enhanced uptake of the radiotracer in myocardium following DOX treatment. While beneficial, ^99m^Tc-incorporated SPECT imaging agents have also inherent limitations, including the continuing threat of serious shortages of ^99m^Mo/^99m^Tc-generators [26, 27]. Additionally, current SPECT tracers also suffer from shortcomings in pharmacokinetics, redistribution of the radiotracer to non-targeted tissues over time, non-linearity of uptake at elevated blood flow (the “roll-off” phenomenon), and low sensitivity [28]. By comparison, positron emission tomography (PET) imaging provides technical advantages, including higher spatial resolution, improved attenuation correction, and the capability to perform quantitative measurements at the peak of stress [29]. Herein, we demonstrate that myocardial uptake profiles of ^68^Ga-Galmydar could provide the earliest indication of DOX-induced cardiomyopathy *in vivo*, while also assessing correlations of myocardial radiotracer uptake with status of mitochondrial potential, using optical imaging at a single cell level. Overall, these data indicate that ^68^Ga-Galmydar could provide a potential diagnostic tool for noninvasive assessment of DOX-induced myocardial dysfunction, while also enabling monitoring of oxidative-stress induced apoptosis.

## Material and methods

All reagents were purchased from Sigma-Aldrich (St. Louis, MO), unless otherwise stated. ^1^HNMR and proton-decoupled ^13^C NMR spectra were recorded on either 300MHz or 400MHzspectrometer (Varian); chemical shifts are reported in δ (ppm) with reference to TMS. Mass spectra were obtained from the Washington University Resource for Biomedical and Bioorganic Mass Spectrometry using samples diluted in 50/50 methanol/water containing 0.1% formic acid and analyzed via HRESI. Elemental analyses were performed by Galbraith Laboratories, Knoxville, TN. HPLC analysis was performed with a Waters System 600 equipped with dual λ-detector 2487 (280 and 214 nm) and a γ-detector (Bioscan) for identification of radiopeaks. Galmydar and its ^68^Ga-radiolabeled counterpart were assessed for purity on a C-18 reversed-phase column (Vydac TP, 10 μm, 300 Å) using an eluent gradient of ethanol and saline (20% ethanol in saline from 0–5 min, 20–90% ethanol in saline from 5–25 min, 90% ethanol in saline from 25–30 min, 100% ethanol from 30–35 min, and 100% saline from 35–40 min; at a flow of 2 mL/min). Radiochemical purity was also determined on C-18 plates, employing a mobile eluent mixture of 90/10 ethanol/saline, using a radio-TLC (Bioscan System 200 Image Scanner).

### Chemistry

The precursor heptadentate ligand, unlabeled Galmydar, and ^68^Ga-Glamydar were synthesized as described earlier [20] and with slight modifications, and are briefly described below:

#### Chemical synthesis

For characterization of the radiotracer, the unlabeled Galmydar was synthesized by treating the precursor heptadentate ligand dissolved in ethanol with a dropwise addition of gallium(III) acetylacetonate dissolved in ethanol, involving the ligand exchange reaction described earlier, and spectroscopically and analytically characterized[20]. ^1^H NMR (300 MHz, DMSO-*d*_6_) δ: 0.79 (s, 6H), 0.96 (s, 6H), 1.30–1.33 (dd, 12H), 2.63 (d, 2H), 2.79 (d, 4H), 2.94 (br, s, 2H), 3.61–3.75 (m, 4H), 4.63 (sept, 2H), 4.79 (br, s, 2H), 6.62 (t, 2H), 6.87 (d, 2H), 7.04 (d, 2H), 8.18 (s, 2H); ^13^C NMR (75 MHz, DMSO-*d*_6_) δ: 22.0, 22.1, 22.2, 26.2, 35.6, 47.7, 59.2, 68.9, 69.5, 115.7, 119.2, 119.5, 125.8, 148.7, 158.1, 170.3; MS (HRESI) Calcd for [C_32_H_48_N_4_O_4_Ga]^+^: 621.2926, found: *m*/*z* = 621.2930; and Calcd for [^13^C_32_H_48_N_4_O_4_Ga]^+^: 622.2959, found: *m*/*z* = 622.2967. Elemental analysis calculated for C_32_H_48_N_4_O_4_Ga+CH_4_O: C 50.72; H 6.71; N 7.17; Ga 8.92%. Found: C 50.51; H 6.68; N 7.08; Ga 9.05%.

#### Radiochemistry

Radiolabeled ^67/68^Ga-Galmydar was synthesized using a procedure described earlier [20] with slight modifications. Briefly, ^68^Ga was eluted from a generator (Eckert & Ziegler Eurotope; IGG100-50M) using 0.1M HCl, the eluted mixture was passed through cation exchange column (Phenomenex; Strata-X-C 33 μm Polymeric Strong Cation; 30 mg/mL) to remove tracer metal impurities, and finally pure ^68^Ga (370–444 MBq) was eluted with 400 μL of 0.02 M HCl in 98/2 acetone/water [30]. Thereafter, HEPES buffer (pH 5.45, 400 μL) was added to the eluent mixture, the pH was adjusted to 4.5, mixed with a solution of the heptadentate Schiff-base precursor ligand (50μg) dissolved in ethanol, and heated at 100°C for 20 min. The reaction was monitored using radio-TLC. Following completion of the reaction, the reaction mixture was diluted with water (5mL), and pH was adjusted to 7 using 10% NaOH (5–10 μL). Finally, ^68^Ga-Galmydar was purified using a C-18 reversed-phase column, employing the gradient eluent mixture of ethanol and saline (as described above), using radio-HPLC and also analyzed using radio-TLC (methanol/saline (90/10); Rf = 0.9; >95% purity; radio-HPLC: Rt = 17.0 min, radiochemical yield: 222–296 MBq (A_m_: 358–477 MBq/nM), 60%). The radiotracer was characterized by spiking an unlabeled analytically characterized sample of Galmydar (10 μg) with the ^67/68^Ga-radiolabeled counterparts, using UV and radio-detectors. The radiolabeled fraction was concentrated, reconstituted in sterile saline containing 2% ethanol, and employed for micro-PET imaging and post imaging biodistribution studies.

### Bioassays

#### MicroPET imaging and biodistribution studies

**MicroPET/CT Imaging**. All animal procedures were approved by the Washington University Animal Studies Committee. Imaging and biodistribution studies were performed in Sprague-Dawley rats. Five days prior to imaging/biodistribution studies, rats (sex, n = 3) were treated either with Doxorubicin at a dose of 15 mg/kg) or vehicle (saline). Prior to PET imaging, rats were anesthetized with isoflurane (2.0%) via an induction chamber and maintained with a nose cone. Following anesthesia, the rats were secured in a supine position and placed in an acrylic imaging tray. Following 60 min post intravenous tail-vein administration of ^68^Ga-Galmydar (250 μL; 2% ethanol in saline, 5–5.5 MBq), micro-PET static Images were acquired, using a Focus 220 microPET or Inveon PET/CT scanner (Siemens Medical Solutions). PET imaging consisted of a 10-min acquisition. PET data were stored in list mode, and reconstruction was performed using a 3D-OSEM method with detector efficiency, decay, dead time, attenuation, and scatter corrections applied. For anatomical visualization, PET images were also co-registered with CT images from an Inveon PET/CT scanner. ROIs were drawn over the myocardium, and standard uptake values (SUV) were calculated as the mean radioactivity per injected dose per weight.

**Biodistribution Studies**. All animal procedures were approved by the Washington University Animal Studies Committee. Following microPET/CT imaging, rats were sacrificed by cervical dislocation. These studies were done in non-perfused rats. Blood samples were obtained by cardiac puncture, organs then harvested rapidly, and all tissue samples analyzed for γ-activity using Beckman Gamma 8000 counter. All samples were decay-corrected to the time, the γ- counter was started. Standard samples were counted with the organs for each animal, and represent 1% of the injected dose. An additional dose was diluted into milliQ water (100mL) and aliquots (1mL) were counted with each mouse. Data were quantified as the percentage injected dose (%ID) per gram of tissue (tissue kBq (injected kBq)^–1^ (g tissue)^–1^ x 100).

**Cell Culture**. Rat cardiomyoblasts (H9c2(2–1)) cells were grown in Dulbecco’s Modified Eagle Medium (DMEM) supplemented with L-glutamine (2 mM), penicillin/streptomycin (200 I.U.) and heat-inactivated fetal calf serum (10%). The cells were grown at 37°C under a 5% CO_2_ atmosphere.

**Fluorescence Imaging Studies**. For dose-dependent doxorubicin treatment studies, H9c2 cells were plated onto borosilicate 8-well chambered coverglass (Labtek) and allowed to grow to approximately 70% confluence at 37°C under 5% CO_2_ atmosphere in 200 μL of culture medium. For assessment of DOX dependent effects, cells were incubated with concentrations of doxorubicin (0 μg/mL, 1 μg/mL, 5 μg/mL, and 10 μg/mL) for 24 h at 37°C under continuous influx of 5% CO_2_. For evaluating impact of electronegative transmembrane gradients on uptake profiles of the metalloprobe, cells were also incubated with the Galmydar (20 μM) for 1h at 37°C either in presence or absence (control) of 130 mM K^+^ and valinomycin (1μg/mL) in the media. Prior to fluorescence imaging, cells were incubated with Galmydar (20 μM) for 1h at 37°C, while maintaining continuous influx of 5% CO_2_. Cellular accumulation of the Galmydar was assessed using a Nikon Ti-E PFS inverted microscope equipped with a Nikon 40 × 0.3 NA Plan APO objective, Prior H117 ProScan flat top linear encoded stage, and Prior Lumen 200PRO illumination system with standard DAPI (E_ext_: 350 nm; E_em_: 470 nm): and FITC (E_ext_: 495 nm; E_em_: 519 nm) filter sets. Images were acquired using a Photometrics CoolSNAP HQ2 digital camera and MetaMorph Microscopy and Imaging Analysis Software (version 7.7.0.0, Molecular Devices). Images were processed and analyzed using the ImageJ software package (NIH). Following incubation with Galmydar, cells were visualized and images were acquired using appropriate filter sets (DAPI excitation and FITC emission). Cellular uptake of Galmydar was then quantified (wherein corrected total cellular fluorescence (CTCF) = integrated density–(area of selected cell × mean fluorescence of background readings) [31] using protocols described elsewhere [21, 32].

For evaluating a time-dependent Doxorubicin treatment effect, prior to imaging, cells were incubated with Galmydar (20 μg/mL) following treatments with concentrations of DOX (0 μg/mL, or 10 μg/mL). Cellular uptake of Galmydar was then monitored using fluorescence microscopy over the course of 5 h, and then quantified using protocols described earlier. For evaluation of Dox-induced superoxide production, H9c2 cells were incubated with DOX (1μg/mL) for 1h, the extracellular volume was removed, rinsed with PBS (2 × 200 μL), and fresh media (200 μL) was added. Thereafter, MitoSox (0.5 μM) was added, and cells were incubated at 37°C for 15 min. Following incubation, the extracellular volume was removed, rinsed with PBS (2 × 200 μL), and fresh media (200 μL) was added, thereafter images were acquired, and cellular uptake was quantified as described above.

**Radiotracer Bioassays**. Radiotracer uptakes in H9c2 cells were performed in 24-well tissue culture treated plates. Cells (100,000/well) were plated in media and allowed to recover overnight. Media was removed from cells and replaced with media containing the desired concentrations of ^67^Ga-Galmydar (74 kBq/mL) using control buffer either in the absence or presence 130 mM K^+^/valinomycin (1 μg/mL) buffer [33]. Cells were incubated under normal incubation conditions (37°C, 5% CO_2_ atmosphere) for 60 min, and then washed 3× with cold (4°C) phosphate buffered saline (without CaCl_2_ and MgCl_2_). Cells were then extracted in 1% sodium dodecyl sulfate with 10 mM sodium borate. Aliquots of the loading solution and ^67^Ga-Galmydar stock solutions also were obtained for standardizing cellular data with the extracellular concentration of the tracer. All cell extracts, ^67^Ga-Galmydar stock solutions, and loading solution samples were assayed for γ-activity in a well-type sodium iodide γ-counter (Cobra II; Packard). Protein mass was estimated by the bicinchoninic acid analysis (Pierce Chemical Co.), using bovine serum albumin as the protein standard. Data are reported as fmol ^67^Ga-Galmydar (mg protein)^−1^ (nM_0_)^−1^ as previously described [20, 30, 34, 35] with nM_0_ representing the total concentration of ^67^Ga-complex in the extracellular buffer.

**Detection of caspase-3 activation**. Caspase-3 activation was measured using the Alexa Fluor 647 Rabbit Anti-Active Caspase-3 monoclonal antibody (clone C92-6505, BD Biosciences, San Jose, CA) [36]. Both Dox dose- and time-dependent Caspase activation studies were performed.

For dose-dependent caspase-3 activation studies, H9c2 cells were grown in T150 flasks and treated with either 0, 1, 5, or 10 μg/mL of doxorubicin for 24 h. Cells were harvested and washed twice using cold PBS. Cell pellets were resuspended in PBS and stained with a LIVE/DEAD fixable yellow dead cell stain (Thermo Fisher, Carlsbad, CA). Cells were washed twice in PBS, fixed and permeabilized using a BD Cytofix/Cytoperm Kit (BD Biosciences, San Jose, CA) followed by staining with an Alexa Fluor 647 Rabbit Anti-Active Caspase-3 monoclonal antibody. Samples were analyzed on a flow cytometer (Beckman Coulter Gallios) and data were analyzed using FlowJo software (TreeStar, Ashland, OR).

For time-dependent caspase-3 activation studies, H9c2 cells were grown in T150 flasks and treated with either concentrations of 0 or 10 μg/mL of doxorubicin for 1 h and then allowed to recover for 0–4 days. Caspase activation activity over 0–4 days was evaluated with the flow cytometer (Beckman Coulter) and quantified as described above.

**Statistical Analysis**. This imaging study is a proof-of-the concept preliminary study. Due to limitations of small sample size in this feasibility study, nonparametric test were used for testing significance of reported data. Wilcoxon or Kruskall-Wallis tests were deployed to examine differences between groups and Friedman’s Chi-square was used to assess differences within and between groups, as needed. SASv9.4 was used to conduct data analysis (SAS Institute, Cary, NC).

## Results and discussion

Doxorubicin has been known to act as an antitumor agent primarily by intercalation into DNA and inhibiting topoisomerase II (TOP2) in fast-proliferating cancer cells, thus inducing cancer cell death [37]. Importantly, DOX-induced cardiotoxicity involves apoptosis or other forms of cell death in cardiomyocytes, resulting in loss of functional myocytes and irreversible heart injury. However, the mechanism for DOX-mediated cardiotoxicity appears to be separable from its therapeutic mode of action due to 2 factors: a) cardiomyocytes are generally not replicative, and b) Top2α, the primary target of doxorubicin, is not expressed in quiescent cells, and is undetectable in heart tissues [38, 39]. Therefore, various mechanisms for DOX-induced cardiomyopathy have been proposed, such as generation of reactive oxygen species (ROS) [40, 41], impaired mitochondrial function, disruption of Ca^2+^ homeostasis, and altered gene and protein expression that triggers cell death [42, 43]. Furthermore, it has also been shown that changes in mitochondrial redox state, and membrane potential could precede DOX-induced cell death [44, 45].

**Fig 2:**
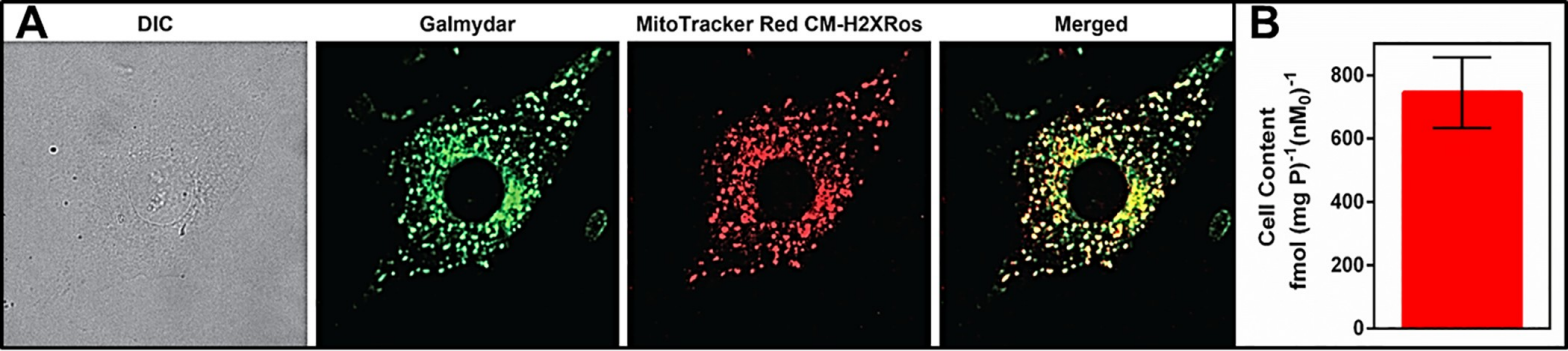
(A) **Cellular accumulation of Galmydar and demonstration of its intracellular localization in mitochondria via correlation with Mito-Tracker:** Images were acquired using a 60 × objective (all panels represent same magnification) in live H9c2(2–1) cells following 30 min treatment with Galmydar (20 μM) and MitoTracker Red CM-H2XRos (25 nM). **A**: DIC; **B**: Uptake of Galmydar; **C**: Uptake of MitoTracker Red; **D**: Merge of **B** & **C**. (B) **Cellular accumulation of** ^**67**^**Ga-Galmydar in rat cardiomyoblasts H9c2(2–1)**. Shown is a net uptake at 90 minutes (fmol nM_0_^−1^ mg protein^−1^) using the control media. The bar represents the mean of 4 determinations; the line above the bar denotes ±SD.

Previously, we reported that Galmydar is a fluorescent probe, and its uptake within human breast carcinoma cells is a net function of transporter-mediated efflux at plasma membrane and negative transmembrane electrochemical gradients-mediated influx present at plasma- and mitochondrial membranes (**Fig 2**)[21]. To assess its accumulation profiles, and intracellular localization at a single cell level, Galmydar was incubated with rat cardiomyoblasts (H9c2) either in presence or absence of mitotracker Red CM-H2XRos (25 nM; a well- validated positive control for mapping mitochondria) [46–48] at 37°C for 60 min, and uptake was evaluated, using live cell imaging. Galmydar penetrated cells and showed stable retention within H9c2 cells. Importantly, the localization of Galmydar within mitochondria of H9c2 cells also mapped closely with that of mitotracker Red **(Fig 2A)** thereby confirming its intracellular localization within mitochondria of H9c2 cells. To assess whether or not, cellular accumulation profiles using a fluorescent read out are in accord with that of radiotracer bioassay, ^68^Ga-Galmydar was also incubated in H9c2 cells at 37°C for 60 min, and the cell uptake was quantified using methodologies described earlier. While the optical imaging allows investigations of uptake profiles for the metalloprobe at a single cell level, the radiotracer assay enables quantification of accumulation of a given probe in population of cells under identical conditions. To assess radiotracer uptake profiles, ^68^Ga-Galmydar was incubated in H9c2 cells for 60 min, and the radiotracer uptake was quantified by normalizing the counts to cell protein. Importantly, ^68^Ga-Galmydar permeated H9c2 cells, demonstrated very high levels of the radiotracer uptake (745±112 fmol (mg P)^−1^ (nM_0_)^−1^), and the radiotracer uptake profiles match fairly well with that of an optical readout **(Fig 2B)**. In the absence of Pgp expression in H9c2 cells, ^68^Ga-Galmydar uptake could also be beneficial in deducing mitochondrial function. Therefore, we postulate that ^68^Ga-Galmydar could likely provide an earliest readout about the status of mitochondrial potential and resultant downstream impact on initiation of apoptosis *in vivo*.

**Fig 3:**
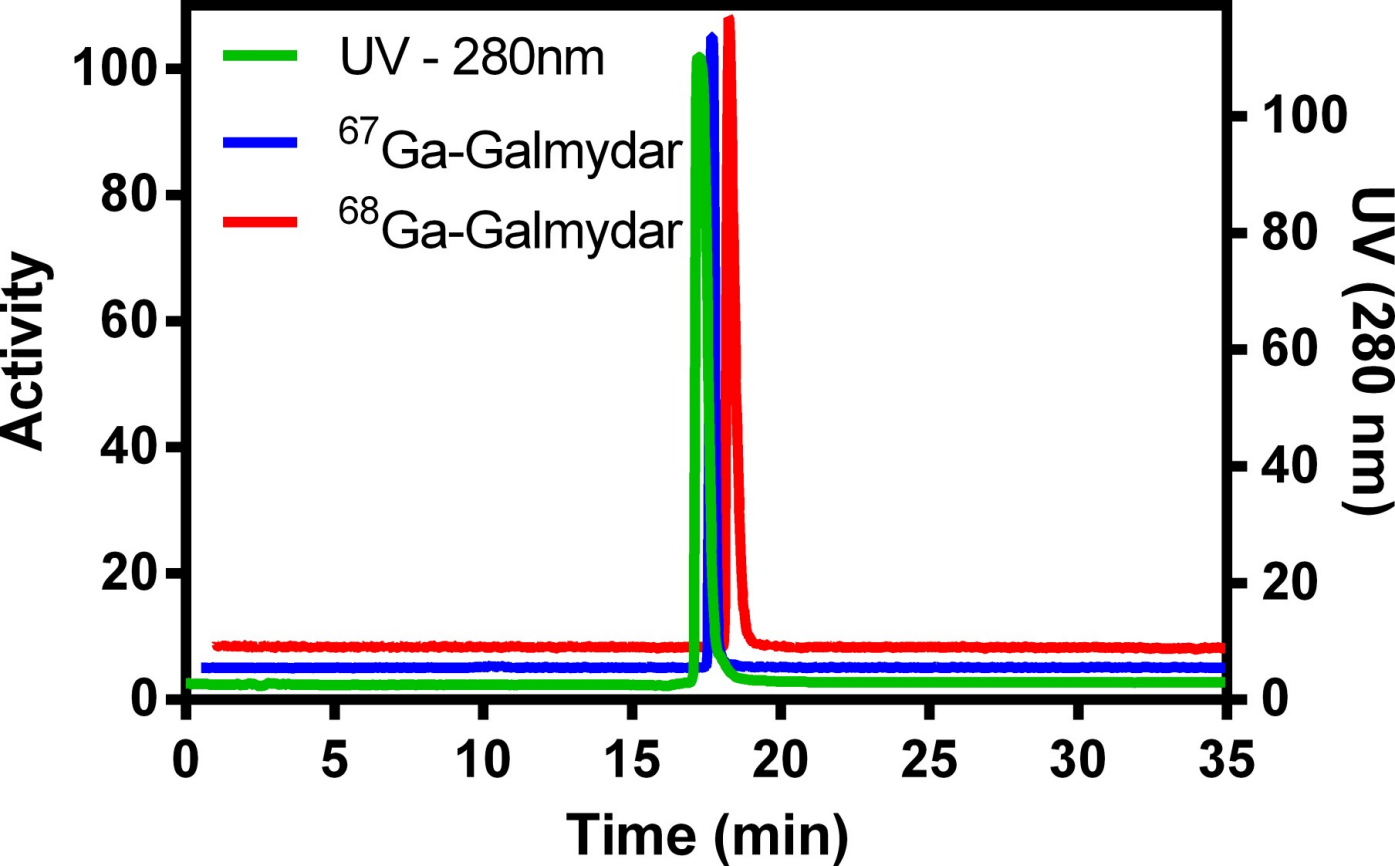
Radio-HPLC data for ^67^Ga-Galmydar (Blue) and ^68^Ga-Galmydar (Red) spiked with an unlabeled Galmydar (Green). The peaks are off-set for clarity.

**Fig 4:**
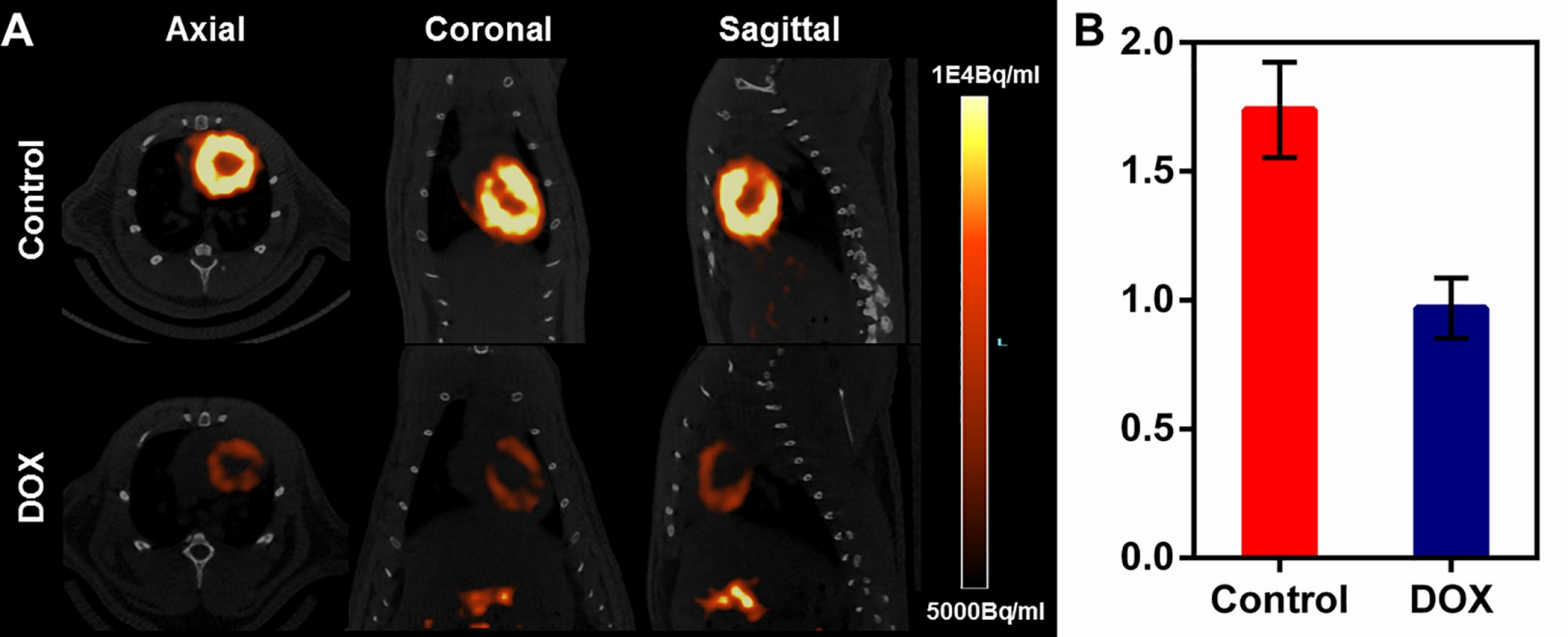
(A) **Micro-PET/CT Imaging**. Sprague-Dawley rats were injected intravenously with ^68^Ga-Galmydar and static PET images were acquired for 10 min, 60 min post tail-vein injection. Top Panel: Control Rat; Lower Panel: DOX (15mg/kg, 5 days prior to imaging)-treated Rat. Similar results were obtained in 3 independent experiments. (B) SUV analysis of ^68^Ga-Galmydar uptake in hearts of SD rats (mean ± SD, n = 3).

**Fig 5:**
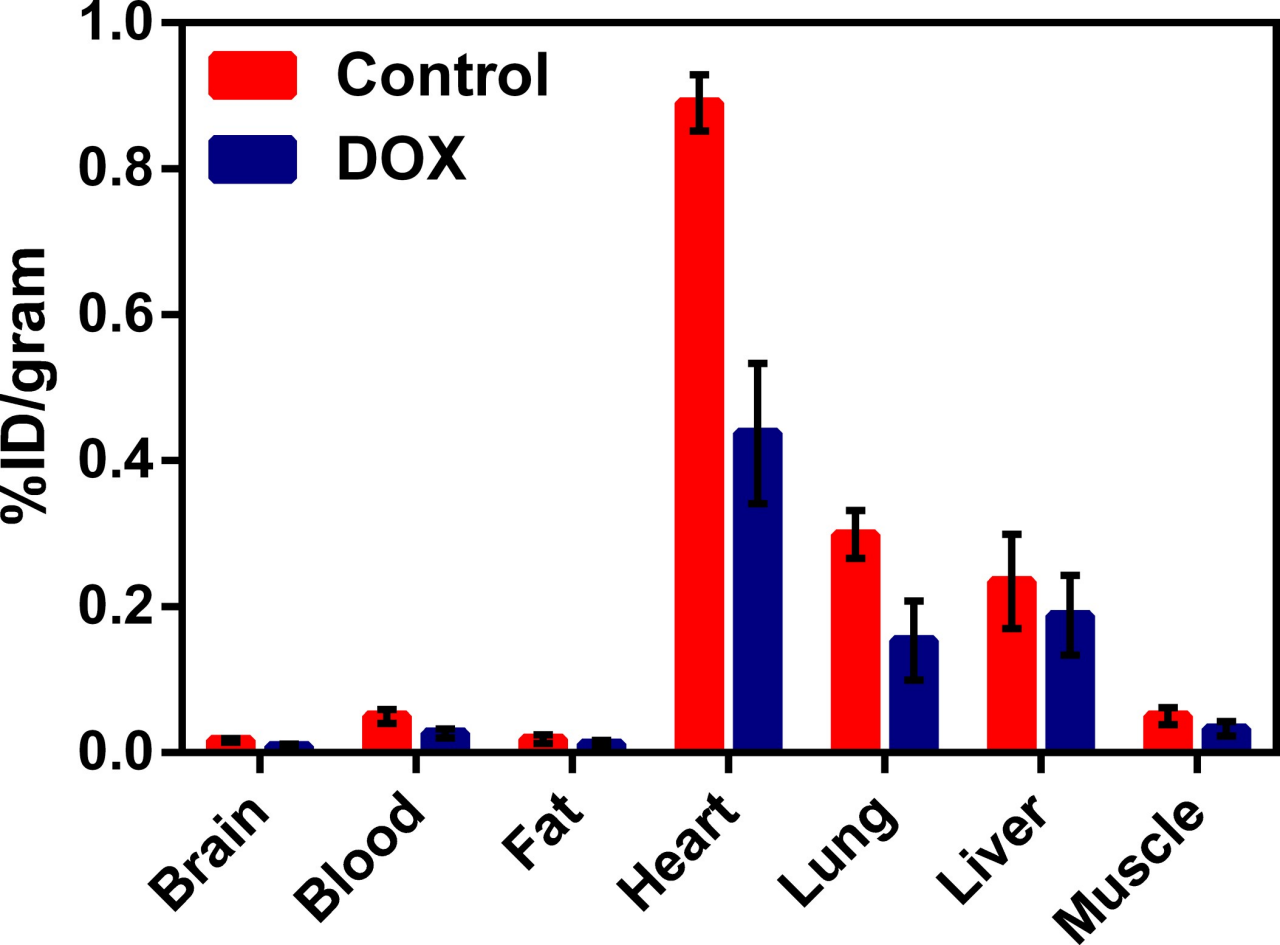
Post-imaging biodistribution data (%ID/g) for ^68^Ga-Galmydar in rats treated either with DOX (15mg/kg; 5 days prior to imaging) or vehicle as a control (mean ± SD, n = 3). Using Wilcoxon 2-sample tests, and one-sided, the p values were obtained for uptake in each organ (Brain: z = 1.75, p = 0.07; Blood: z = 1.74, p = 0.04; Fat: z = 1.31, p = 0.10; Heart: z = 1.75, p = 0.04; Lung: z = 0.44, p = 0.34; and muscle: z = 1.31, p = 0.10).

Earlier, we have shown (through quantitative pharmacokinetic studies) that ^68^Ga-Galmydar penetrates myocardium of mice and rat, minutes post tail-vein injection. While rapidly excreting from blood pool within minutes to nearly background levels, ^68^Ga-Galmydar also indicated a high extraction and stable retention in myocardium of mice and rat as a function of time (5 min through 2 h) post tai-vein injection [20]. These data indicated the potential utility of ^68^Ga-Galmydar PET imaging to enable interrogation of heart function *in vivo*. Additionally, TACs from dynamic PET scans (following tail-vein injections of the radiotracer) showed heart uptake and stable retention in rats as a function of time (up to 70 min, data not shown). To further assess potential of ^67/68^Ga-Galmydar to serve as a molecular imaging probe for assessment of DOX-induced cardiotoxicity *in vivo*, ^67/68^Ga-Galmydar was synthesized through a ligand-exchange reaction using procedures described earlier [20], purified, and analyzed using radio-HPLC, **Fig 3**). Finally, ^67/68^Ga-Galmydar was reconstituted in saline containing ethanol (2%), and deployed for either cell uptake assay or microPET/CT imaging experiments. Prior to imaging, rats were pretreated either with intravenous administration of DOX (15 mg/kg) or vehicle (5% ethanol in saline) for 5 days. Following treatments, micro-PET static scans (10 min acquisition; 60 min post tail-vein administration of ^68^Ga-Galmydar; **Fig 4A**) demonstrated a 1.91-folds lower retention in hearts of DOX-treated (Standard Uptake Value; SUV: 0.92, n = 3) rats compared with their vehicle treated counterparts (SUV: 1.76, n = 3), (**Fig 4B**). For confirmation of PET data, post-imaging quantitative biodistribution studies were also performed. All rats were sacrificed by cervical dislocation, organs excised, counted for γ activity, and the activity was normalized to % injected dose (**ID**) and weight. The post imaging pharmacokinetic data demonstrated heart retention values of 2.02 folds lower for DOX treated (%ID/g; DOX: 0.44 ± 0.1, n = 3) rats compared to their vehicle treated counterparts (Vehicle Control: 0.89 ± 0.03, n = 3, p = 0.04), **Fig 5**, thus further supporting micro-PET imaging data *in vivo*.

The normal function of mitochondria is essential for controlling inter-dependent biochemical pathways, such as apoptotic signaling, intracellular Ca^+2^ regulation, and ROS generation. Although the exact mechanism (s) of DOX-induced cardiotoxicity have been intensely studied, the formation of free-radicals following binding to iron continues to be the main mode of action [49]. Other mechanisms involve direct DNA damage, blocking of mitochondrial adenine-triphosphate generation, and release of apoptotic proteins (caspases). Importantly, myocardium is more susceptible to free-radical damage compared with other tissues due to less abundance of free-radical scavenging enzymes (superoxide dismutase and catalase) in addition to suppression of glutathione peroxidase [50]. Over the past decades, the degeneration of mitochondria has also been attributed to modification of multi-molecular complex (mitochondrial permeability transition pore, mPTP), a high conductance channel in the inner membrane of mitochondria, which is permeable to solutes up to 1.5 kDa. Overall, the mPTP is a Ca^+2^, pH, redox-, and voltage sensitive channel. Thus opening of the mPTP can either be transient thus a reversible event or a long-lasting process. While the transient opening regulates critical physiological processes, such as intracellular Ca^+2^ homeostasis, NAD^+^ trafficking, and production of transient ROS[51, 52], it is typically not associated with cell death. However, the long-lasting opening of mPTP is normally considered irreversible, thus increases mitochondrial permeability to ions, and solutes, and resultant loss of electrochemical gradients, thereby leading to depolarization of mitochondrial potential, reduction in ATP levels, increase in ROS production, Ca^+2^ release, vacuolization and swelling of mitochondria thus eventually mediating progression to cell death [53–55]. Overall, these observations indicate that DOX-mediated activity likely result in a depolarization of mitochondrial potential and subsequent caspase activation [22].

**Fig 6:**
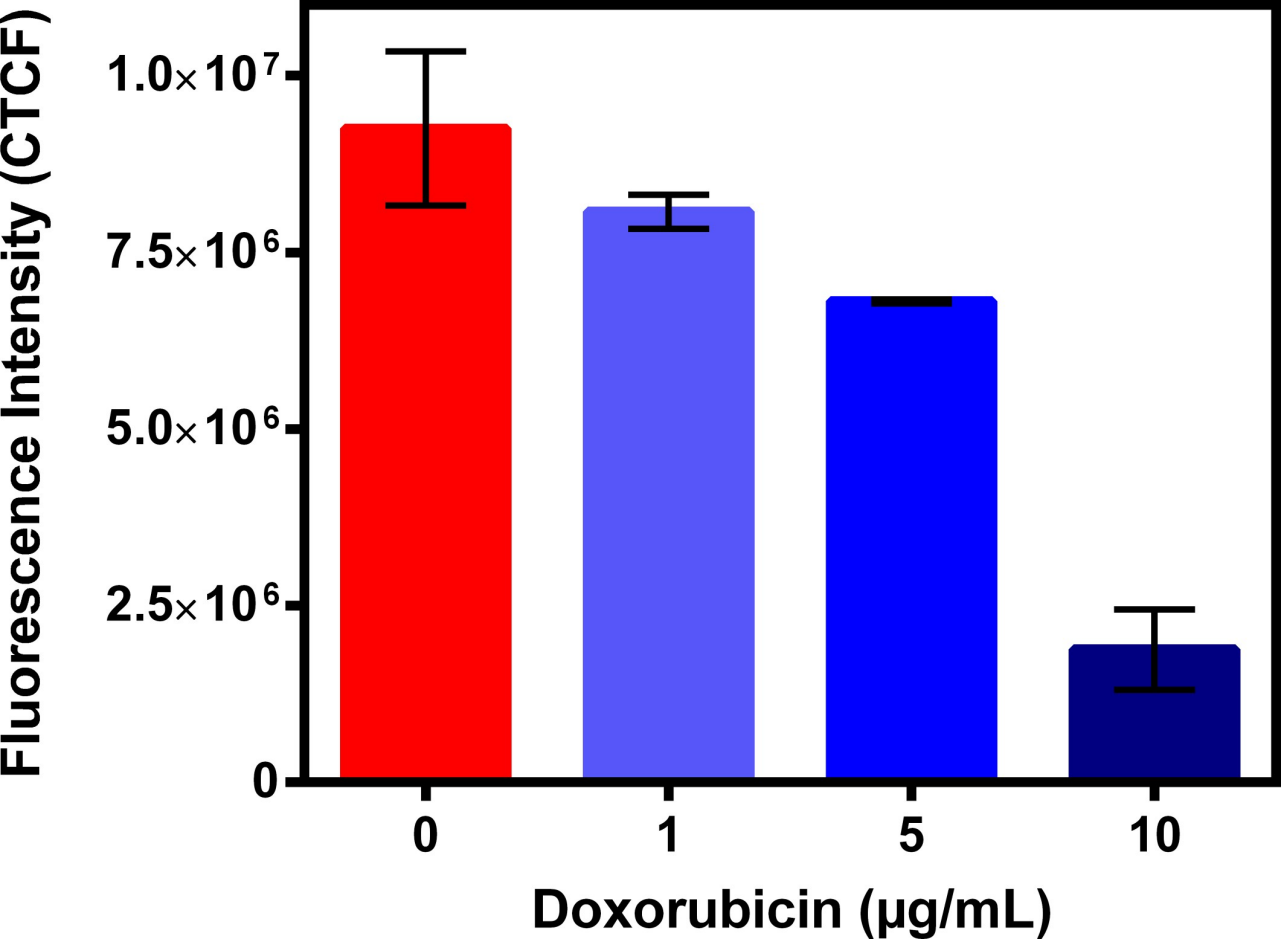
DOX induced cardiomyopathy in rat cardiomyocytes cells. H9c2 cells were treated with DOX (0, 1, 5 and 10 μg/mL) for 24h and uptake of Galmydar (20 μM, 60 min) was determined using fluorescence imaging. Corrected Total Cell Fluorescence (CTCF) = Integrated Density–(Area of selected cell X Mean fluorescence of background readings); (mean ± SEM, *H* (3, N = 3) 9.45, p = 0.02, Kruskal-Wallis).

**Fig 7:**
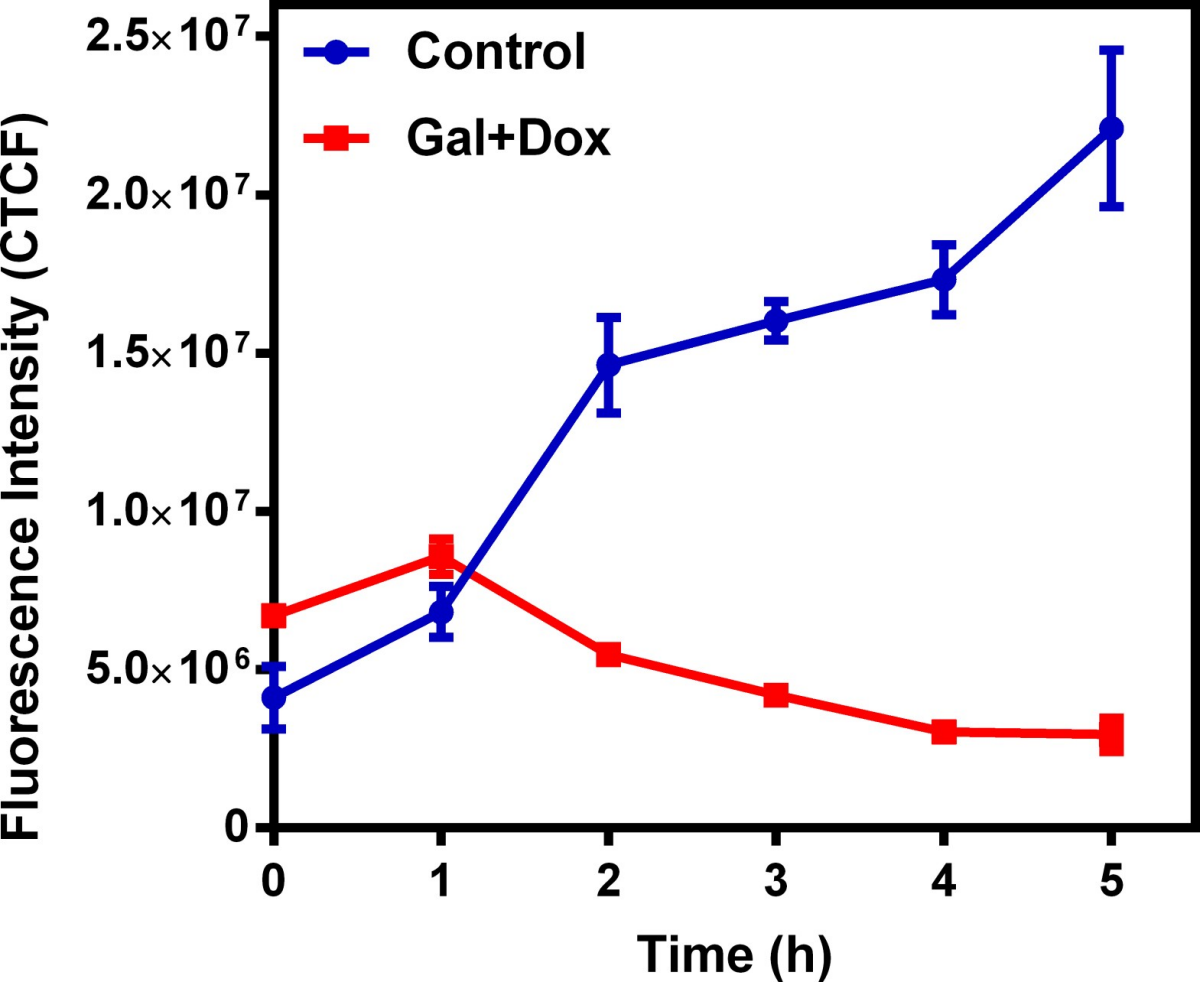
DOX induced cardiomyopathy in rat cardiomyocytes cells. H9c2 cells were treated with DOX (0 and 10 μg/mL) for 0–5 h and uptake of Galmydar (20μM, 60 min) was determined using fluorescence imaging. Corrected Total Cell Fluorescence (CTCF) **=** Integrated Density–(Area of selected cell × Mean fluorescence of background readings); (mean ± SEM, χ_r_^2^ (1, N = 35) = 2.88, p = 0.09, Friedman’s chi-square).

**Fig 8:**
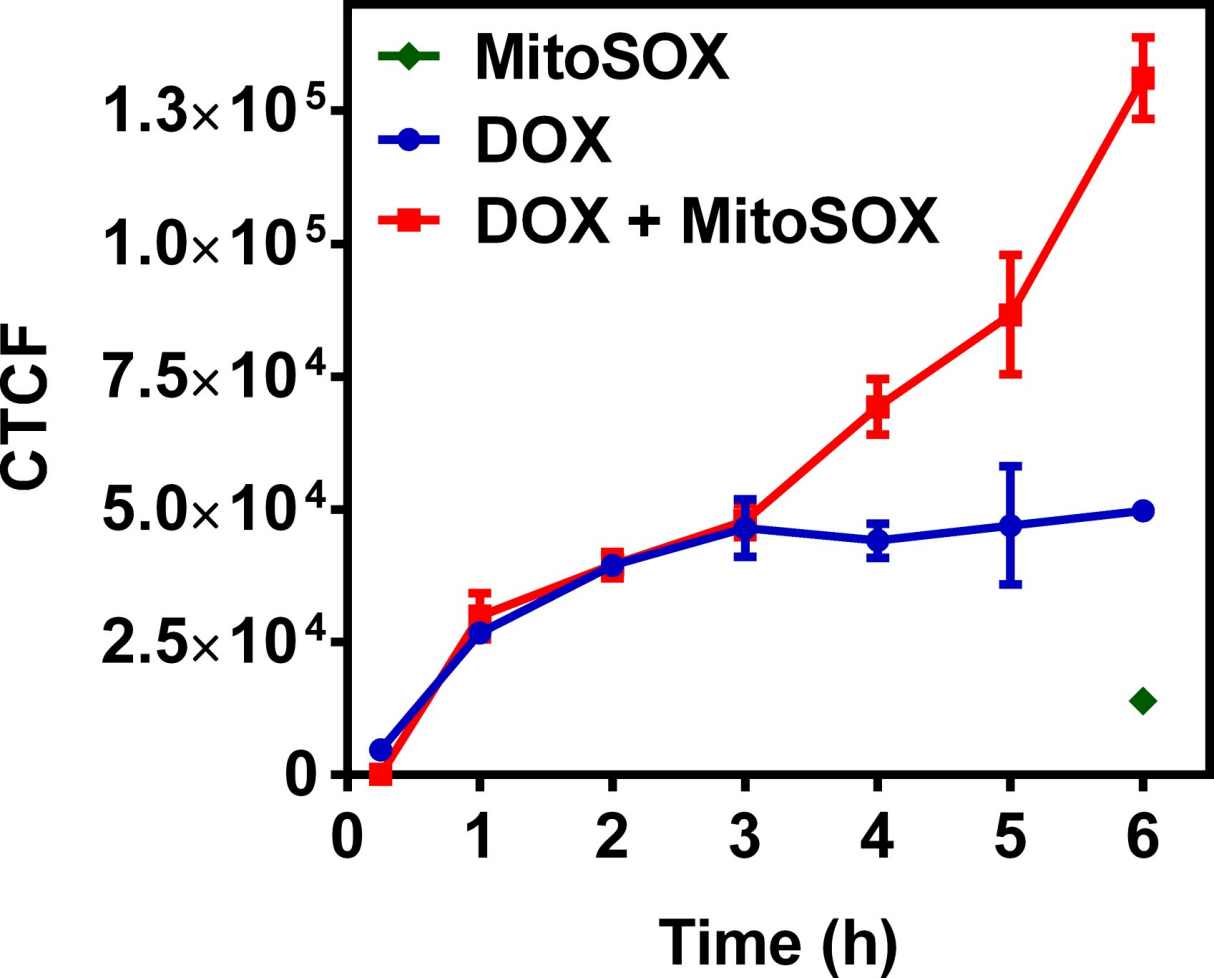
DOX induced superoxide production in rat cardiomyocytes cells. H9c2 cells were treated with DOX (1μg/mL) for 60 min at 37°C, extracellular space was rinsed with PBS (2 times), replaced with media, incubated with MitoSox (0.5μM) at 37°C for 15 min, and intracellular uptake was analyzed using fluorescence imaging. Corrected Total Cell Fluorescence (CTCF) **=** Integrated Density–(Area of selected cell × Mean fluorescence of background readings); (mean ± SEM; p > 0.01).

**Fig 9:**
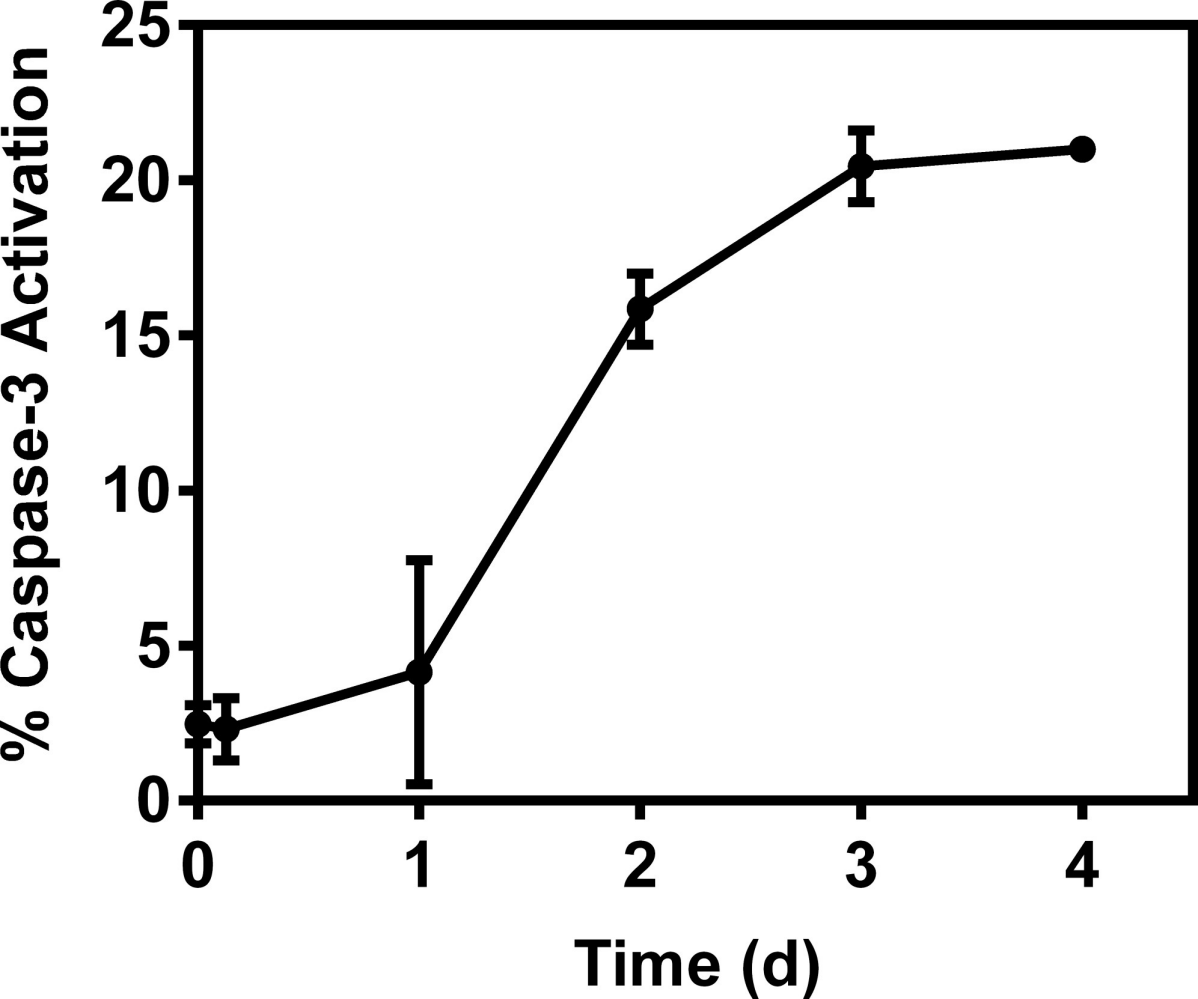
DOX induced caspase-3 activation in rat cardiomyocytes cells. H9c2 cells were treated with DOX (0 and 10 μg/mL) for 1h, recovered for 0–4 days and caspase-3 activation was determined using flow cytometry (χ_r_^2^ (1, N = 11) = 7.47, p = 0.19, Friedman’s chi-square).

To further understand possible biochemical processes driving the resulted decreased retention of the radiotracer within the rat myocardium, the cellular accumulation of Galmydar was performed in cultured H9c2 rat cardiomyoblasts. For cell uptake evaluations, both radiotracer- and live-cell fluorescence imaging [21, 32] studies were performed. To accomplish this objective, we evaluated uptake profiles of Galmydar in cultured H9c2 rat cardiomyoblasts using fluorescence microscopy, and subsequent quantification was performed. Of note, Galmydar is a fluorescent molecule (E_ex_: 375 nm, E_emis_ = 485 nm) [30]. Because DOX causes dose-dependent cardiomyopathy in cancer patients[40], we evaluated cell uptake of Galmydar as function of DOX concentration, using live cell fluorescence imaging. For investigations, H9c2 cells were treated with increasing concentration of DOX (1 μg/mL, 5 μg/mL, and 10 μg/mL)[44] for 24 h at 37°C under a continuous flux of 5% CO_2_. After 24h, cells were recovered in fresh growth media, and then incubated with Galmydar (20 μM) for 1 h at 37°C under 5% CO_2_, and cellular accumulation was analyzed under identical conditions, using a Nikon Ti-E PFS inverted microscope. Galmydar demonstrated cell uptake profiles mediated by DOX-dose (**Fig 6**), wherein probe accumulation deceased as a function of increased DOX-dose (1.0-fold depression in 1μg/mL, 1.3-fold depression in 5 μg/mL, and 5.2-fold depression in 10 μg/mL, *H* (3, N = 3) 9.45, p = 0.02, Kruskal-Wallis). To further evaluate uptake kinetics of Galmydar (20 μM) as a function of time, H9c2 cells were also plated and incubated with DOX or vehicle at 37°C under a continuous flux of 5% CO_2_, recovered to fresh growth media, immediately treated with Galmydar (20 μM) and monitored for 5 h under identical conditions, using a Nikon Ti-E PFS inverted microscope. Over the course of 5 h, the cultured rat myocardium cells exhibited a gradual depression in cellular uptake and retention of Galmydar (**Fig 7**; up to 8.2-fold difference compared to their untreated cells after 5 h, p = 0.09, Friedman’s chi-square), thus indicating the sensitivity of the probe to map changes at the level of the mitochondria resulting from DOX treatment, which in turn likely result from depolarization of the mitochondrial potential. These findings are consistent with literature precedents, wherein DOX treatment has been shown to alter mitochondrial redox potentials, thus depolarizing mitochondria, and elevating matric Ca^2+^ and ROS production in 30 minutes [15]. Furthermore, the reduction of DOX in the mitochondria results in the production of its semiquinone form, which reacts with iron, oxygen and hydrogen peroxide to produce ROS [15]. It has been postulated that DOX treatments alter mitochondrial redox potentials towards a more oxidized state, depolarize mitochondria, elevate matrix calcium levels, and elevate ROS production. To evaluate, whether ROS production in DOX-treated H9c2 cells is indeed mediated by superoxide production, live-cell imaging was performed, and fluorescence signal was quantified (**Fig 8**) as a function of time following DOX-treatments. MitoSox (a well validated fluorescent probe to monitor superoxide production in mitochondria [56, 57]) signal was enhanced by 1.5–2.6 folds, 4–6 h following Dox-treatments of H9c2 cells (**Fig 8**) thus demonstrating a time-dependent pharmacological response consistent with production of superoxide in the mitochondria. Importantly, while decreased retention of Galmydar was evident through depolarization of the mitochondrial potential after 1h post incubation (**Fig 7**), the superoxide production was found to be observed after 3h post-treatment (**Fig 8**) thus indicating mitochondrial depolarization precedes superoxide production under these conditions. The mitochondria respond to this excessive oxidative stress, depolarization, and altered redox states by formation of a mitochondrial permeability transition pore (MPTP). The MPTP allows Cytochrome *c* to leak out, which triggers a cascade leading to the activation of the effector caspase-3 and thereby inducing apoptosis [15]. To evaluate whether or not Galmydar imaging provides an upstream indication of induction of apoptotic pathways as mitochondria begin to depolarize, flow cytometric assays were also performed. Upon treatment with increasing dosages of doxorubicin, flow cytometry revealed increasing caspase-3 activation, inversely proportional to the Galmydar uptake depression observed in fluorescence microscopy studies. Furthermore, caspase-3 activation was shown to progressively increase over the course of 4 days after doxorubicin treatment, indicating that Galmydar demonstrated sensitivity to map mitochondrial defects that precede the mitochondrial apoptotic pathway involving effector caspase-3 (**Fig 9**). This observation is further supported by the findings from the biodistribution studies, which suggests a possible reduction in Galmydar retention within the rat heart parenchyma following single dose treatment (5 days earlier) of doxorubicin (χ_r_^2^ (1, N = 11) = 7.47, p = 0.19, Friedman’s chi-square).

**Fig 10:**
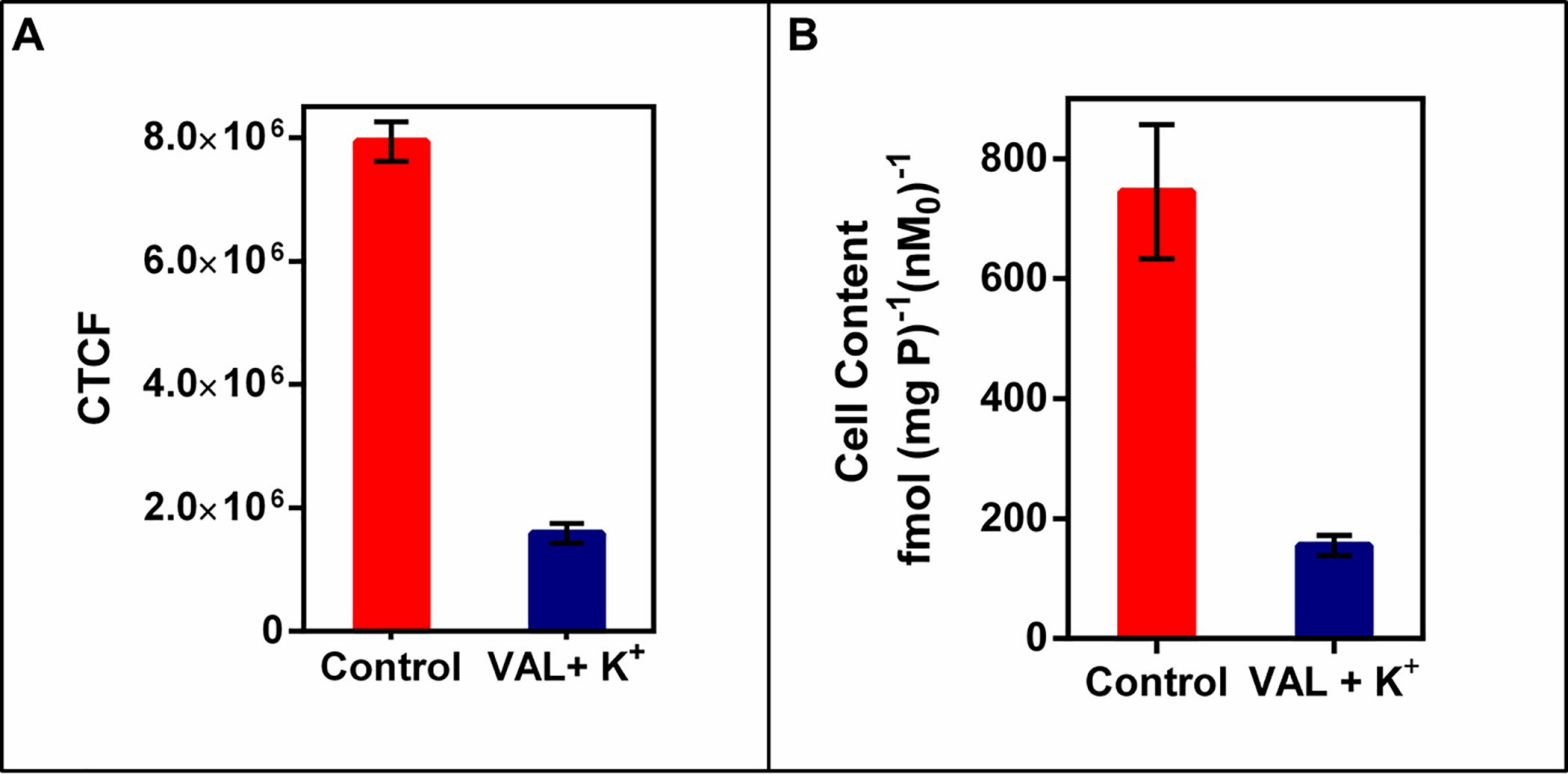
(A) **Cellular accumulation of Galmydar in rat cardiomyoblasts H9c2(2–1) either in control buffer or in presence of 130 mM K**^**+**^ **buffer containing valinomycin (1** μ**g/mL), the potassium ionophore**. Shown is a net uptake at 60 minutes (CTCF) derived from live-cell imaging data. The bar represents the mean of 4 determinations; the line above the bar denotes ±SD. Note dissipation of prevalent electronegative gradients on membranes resulting from influx of K^+^ into cells leading to 80% lower uptake (red bar) compared with that of control (blue) under identical conditions. (B) **Cellular accumulation of** ^**67**^**Ga-Galmydar in rat cardiomyoblasts H9c2(2–1) either in control buffer or in presence of 130 mM K**^**+**^ **buffer containing valinomycin (1** μ**g/mL), the potassium ionophore**. Shown is a net uptake at 90 minutes. Data are reported represented as fmol of ^67^Ga-Galmydar (mg protein)^−1^ (nM_0_)^−1^, wherein nM_0_ representing the total concentration of ^67^Ga-complex in the extracellular buffer. The bar represents the mean of 4 determinations; the line above the bar denotes ±SD. Note uptake profiles of the radiotracer correlate with that of fluorescence live cell imaging.

To further access whether or not the decreased uptake of Galmydar in H9c2 cells is indeed resulting from depolarization of mitochondrial potential at a single cell level, Galmydar was incubated in H9c2 cells in the media either in presence or absence of valinomycin (1μg/mL), an ionophore and high potassium (130 mM KCl) concentration at 37°C for 1h under a continuous flux of 5% CO_2_; and cell uptake was quantified as corrected total cellular fluorescence (CTCF). Valinomycin is a dodecadepsipeptide, and an ionophore which facilitates transfer of K^+^ across the cellular membranes thus resulting in dissipation of both plasma-and mitochondrial electrochemical gradients. Compared with control media (CTCF = 7.94× 10^6^ ± 3.17× 10^5^), Galmydar uptake was reduced (CTCF = 1.6 × 10^6^ ± 1.5× 10^5^) to approximately 20% of control in H9c2 cells following incubation in the media containing valinomycin in high K^+^ concentrations; **Fig 10A** thus indicating a response of the metalloprobe to depolarization of the electrochemical gradients. Similar uptake profiles were also observed using ^67^Ga-Galmydar 155±17 fmol (mg P)^−1^ (nM_0_)^−1^, <20% of control (745±112 fmol (mg P)^−1^ (nM_0_)^−1^), the SPECT counterpart described earlier (**Fig 10B**). Combined data indicate that significant portion of uptake of the metalloprobe is driven by its response to electronegative membrane potentials, and also demonstrates its potential to monitor depolarization of the mitochondrial potential *in cellulo*.

## Conclusions

Following a single dox treatment of rats, microPET/CT imaging shows 2-fold lower retention of ^68^Ga-Galmydar in myocardium compared with their vehicle treated control counterparts. SUV values obtained from micro-PET/CT imaging studies also correlated with myocardium uptake and retention of the tracer using post-imaging quantitative biodistribution studies. Additionally, treatments of myocardial cells with doxorubicin resulted in gradual decrease of Galmydar uptake and retention within mitochondria of H9c2 cells in a dose-dependent and time-dependent manner. Noticeably, this decrease of Galmydar accumulation, and retention in H9c2 cells is also consistent with depolarization of electronegative membrane potentials and activation of caspase-3 activity resulting in doxorubicin-induced cardiotoxicity and cell death. Given that mitochondrial depolarization precedes detection of elevated superoxide production within H9c2 cells, and Galmydar accumulation within these cells is a net function of mitochondrial energetics, these findings suggest that ^68^Ga-Galmydar imaging could potentially provide an early read-out of apoptotic processes, wherein mitochondrial potential undergoes either transient or permanent changes thus leading to impairment in mitochondrial function. Combined data indicate that decreased uptake profiles of the radiotracer observed, following treatments with DOX are in accord both *in vivo* and *in cellulo* profiles at a single cell level; and are likely attributed to impairment of mitochondrial potential[20, 32]. However, these data need to be further ascertained with other functional myocardium viability methodologies using either MRI or US in vivo. While these studies provide preliminary evaluation of ^68^Ga-Galmydar potential to monitor DOX-induced cardiotoxicity 5-d post administration of a single dose, it is also evident from **Fig 8** that significant activation of caspase 3 takes place in H9c2 cells by 2^nd^ day of the DOX-treatment. Therefore, additional micro-PET imaging studies are needed to evaluate sensitivity of ^68^Ga-Galmydar to investigate DOX-induced cardiotoxicity in vivo at earlier time-points, and correlate imaging data with histochemical staining. Although mitochondrial energetics are altered upstream prior to either elevated superoxide production or drug-induced upregulation of caspase-3 activation, it is conceivable that DOX treatments could have some effect on perfusion thus partially contributing to the decreased retention of ^68^Ga-Galmydar in heart, therefore further studies are needed to ascertain contribution of perfusion effects (if any) following DOX treatments, using FDA approved tracer, such as ^11^N-NH_3_. Following further biochemical validations and functional correlations with other imaging modalities such as MRI, ^68^Ga-Galmydar could provide a specific and sensitive noninvasive molecular imaging technique for monitoring mitochondrial impairment, thereby enabling stratification of therapeutic choices for patients undergoing chemotherapeutic treatment in general, and DOX-treatment in particular. Further investigations (in rat and rabbit models) with other chemotherapeutics (both traditional drugs and antibodies) that are susceptible to similar mode of action, and likely to induce cardiotoxicity, are underway.

## Supporting information

S1 Fig NMR Spectra of Galmydar in DMSO-*d*_6_: ^1^H NMR (**Top**); ^13^C NMR (**Bottom**).
(TIF)

S2 Fig Crystal structure of Galmydar showing presence of methanol (also evident in NMR spectra) as a solvate used for calculating elemental percentages in the given mass of Galmydar. (TIF)

S3 Fig High resolution mass spectrum for Galmydar. (TIF)

S1 Table Elemental analysis of Galmydar including methods for quantitative measurements. (DOCX)

S2 Table Theoretical calculations of elements and observed values (%) with errors calculated for analyzed elements. (DOCX)

## References

[1] Frost BM, Eksborg S, Bjork O, Abrahamsson J, Behrendtz M, Castor A, et al Pharmacokinetics of doxorubicin in children with acute lymphoblastic leukemia: multi-institutional collaborative study. Med Pediatr Oncol. 2002;38(5):329–37. Epub 2002/04/30. 10.1002/mpo.10052 . 11979457

[2] Larson RA. Etiology and management of therapy-related myeloid leukemia. Hematology American Society of Hematology Education Program. 2007:453–9. Epub 2007/11/21. 10.1182/asheducation-2007.1.453 . 18024664

[3] Ning YM, He K, Dagher R, Sridhara R, Farrell AT, Justice R, et al Liposomal doxorubicin in combination with bortezomib for relapsed or refractory multiple myeloma. Oncology (Williston Park). 2007;21(12):1503–8; discussion 11, 13, 16 passim. Epub 2007/12/15. . 18077994

[4] Hopkins-Donaldson S, Yan P, Bourloud K, Muhlethaler A, Bodmer J, Gross NJ. Doxorubicin-induced death in neuroblastoma does not involve death receptors in S-type cells and is caspase-independent in N-type cells. Oncogene. 2002;21:6132–7. 10.1038/sj.onc.1205879 12203125

[5] Benson JR, Jatoi I. The global breast cancer burden. Future Oncol. 2012;8(6):697–702. Epub 2012/07/07. 10.2217/fon.12.61 . 22764767

[6] Ferrandina G, Corrado G, Licameli A, Lorusso D, Fuoco G, Pisconti S, et al Pegylated liposomal doxorubicin in the management of ovarian cancer. Therapeutics and clinical risk management. 2010;6:463–83. Epub 2010/10/20. 10.2147/TCRM.S3348 20957139 PMC2952486

[7] Calvo E, Moreno V, Flynn M, Holgado E, Olmedo ME, Lopez Criado MP, et al Antitumor activity of lurbinectedin (PM01183) and doxorubicin in relapsed small-cell lung cancer: results from a phase I study. Ann Oncol. 2017;28(10):2559–66. Epub 2017/09/30. 10.1093/annonc/mdx357 . 28961837 PMC5834091

[8] Matuszczyk A, Petersenn S, Bockisch A, Gorges R, Sheu SY, Veit P, et al Chemotherapy with doxorubicin in progressive medullary and thyroid carcinoma of the follicular epithelium. Horm Metab Res. 2008;40(3):210–3. Epub 2008/03/19. 10.1055/s-2008-1046781 . 18348081

[9] Airoldi M, Cattel L, Milla P, Pedani F, Garzaro M, Dosio F. Paclitaxel and pegylated liposomal doxorubicin in recurrent head and neck cancer: clinical and unexpected pharmacokinetic interactions. Anticancer Res. 2008;28(4C):2519–27. Epub 2008/08/30. . 18751444

[10] Gibb A, Greystoke A, Ranson M, Linton K, Neeson S, Hampson G, et al A study to investigate dose escalation of doxorubicin in ABVD chemotherapy for Hodgkin lymphoma incorporating biomarkers of response and toxicity. Br J Cancer. 2013;109(10):2560–5. Epub 2013/10/19. 10.1038/bjc.2013.605 24136151 PMC3833204

[11] Wagner MJ, Livingston JA, Patel SR, Benjamin RS. Chemotherapy for Bone Sarcoma in Adults. Journal of oncology practice. 2016;12(3):208–16. Epub 2016/03/11. 10.1200/JOP.2015.009944 . 26962160

[12] Vilaseca J, Guardia J, Bacardi R, Monne J. Doxorubicin for liver cancer. Lancet. 1978;1(8078):1367 Epub 1978/06/24. . 78137 10.1016/s0140-6736(78)92448-0

[13] Haas NB, Lin X, Manola J, Pins M, Liu G, McDermott D, et al A phase II trial of doxorubicin and gemcitabine in renal cell carcinoma with sarcomatoid features: ECOG 8802. Med Oncol. 2012;29(2):761–7. Epub 2011/02/08. 10.1007/s12032-011-9829-8 21298497 PMC3566570

[14] Keizer H, Pinedo H, Schuurhuis G, Joenje H. Doxorubicin (adriamycin): a critical review of free radical-dependent mechanisms of cytoxicity. Pharmacol Ther. 1990;47:219–31. 2203071 10.1016/0163-7258(90)90088-j

[15] Lu P. Monitoring cardiac function in patients receiving doxorubicin. Seminars in Nuclear Medici e. 2005;35(3):197–201. Epub 2005/08/16. 10.1053/j.semnuclmed.2005.02.005 . 16098293

[16] Sharma V. Radiopharmaceuticals for assessment of multidrug resistance P-glycoprotein-mediated transport activity. Bioconjug Chem. 2004;15:1464–74. 10.1021/bc0498469 15546216

[17] D'Amore C, Gargiulo P, Paolillo S, Pellegrino AM, Formisano T, Mariniello A, et al Nuclear imaging in detection and monitoring of cardiotoxicity. World journal of radiology. 2014;6(7):486–92. Epub 2014/07/30. 10.4329/wjr.v6.i7.486 25071889 PMC4109100

[18] Sarvazyan N. Visualization of doxorubicin-induced oxidative stress in isolated cardiac myocytes. Am J Physiol. 1996;271(5 Pt 2):H2079–85. Epub 1996/11/01. 10.1152/ajpheart.1996.271.5.H2079 . 8945928

[19] Davies KJ, Doroshow JH. Redox cycling of anthracyclines by cardiac mitochondria. I. Anthracycline radical formation by NADH dehydrogenase. J Biol Chem. 1986;261(7):3060–7. Epub 1986/03/05. . 3456345

[20] Sharma V, Sivapackiam J, Harpstrite SE, Prior JL, Gu H, Rath NP, et al A Generator-Produced Gallium-68 Radiopharmaceutical for PET Imaging of Myocardial Perfusion. PloS one. 2014;9(10):e109361 Epub 2014/10/30. 10.1371/journal.pone.0109361 25353349 PMC4212944

[21] Sivapackiam J, Harpstrite SE, Rath NP, Sharma V. ^67^Ga-metalloprobes: monitoring the impact of geometrical isomers on accumulation profiles in rat cardiomyoblasts and human breast carcinoma cells. Med Chem Commun. 2017;8:158–61. 10.1039/c6md00474a PMC6071725 30108701

[22] Gustafsson AB, Gottlieb RA. Heart mitochondria: gates of life and death. Cardiovasc Res. 2008;77(2):334–43. Epub 2007/11/17. 10.1093/cvr/cvm005 . 18006487

[23] Piwnica-Worms D, Chiu ML, Kronauge JF. Detection of adriamycin-induced cardiotoxicity in cultured heart cells with technetium 99m-SESTAMIBI. Cancer Chemother Pharmacol. 1993;32(5):385–91. Epub 1993/01/01. . 8339390 10.1007/BF00735924

[24] Wakabayashi H, Taki J, Inaki A, Sumiya H, Tuchiya H, Kinuya S. Assessment of doxorubicin cardiac toxicity using 99mTc-MIBI myocardial gated SPECT in patients with malignant. J Nucl Med. 2010;51 (Suppl 2):1720.

[25] Yurekli Y, Unak P, Ertay T, Biber Z, Medine I, Teksoz S. Radiopharmaceutical model using 99mTc-MIBI to evaluate amifostine protection against doxorubicin cardiotoxicity in rats. Annals Nucl Med. 2005;19(3):197–200. 10.1007/BF02984605 15981672

[26] Atcher R. US still vulnerable to isotope shortage. RSNA News. 2011;21(10 &11):9–10.

[27] Gould P. Medical isotope shortage reaches crisis level. Nature. 2009;460(7253):312–3. Epub 2009/07/17. 10.1038/460312a . 19606111

[28] Beller GA, Bergmann SR. Myocardial perfusion imaging agents: SPECT and PET. J Nucl Cardiol. 2004;11(1):71–86. 10.1016/j.nuclcard.2003.12.002 . 14752475

[29] Beller GA. Recent advances and future trends in multimodality cardiac imaging. Heart, lung & circulation. 2010;19(3):193–209. Epub 2010/02/09. 10.1016/j.hlc.2009.11.003 . 20138581

[30] Sivapackiam J, Harpstrite SE, Prior JL, Mattingly S, Sharma V. (67/68)Galmydar: A metalloprobe for monitoring breast cancer resistance protein (BCRP)-mediated functional transport activity. Nucl Med Biol. 2016;43(3):191–7. Epub 2016/03/01. 10.1016/j.nucmedbio.2015.12.001 . 26924499 PMC12369548

[31] McCloy RA, Rogers S, Caldon CE, Lorca T, Castro A, Burgess A. Partial inhibition of Cdk1 in G 2 phase overrides the SAC and decouples mitotic events. Cell Cycle. 2014;13(9):1400–12. Epub 2014/03/15. 10.4161/cc.28401 24626186 PMC4050138

[32] Sundaram G, Sharma M, Kaganov D, Cho JJ, Harpstrite SE, Sharma V. Metalloprobes: Fluorescence imaging of multidrug resistance (MDR1) P-Glycoprotein (Pgp)-mediated functional transport activity in cellulo. J Inorg Biochem. 2016;159:159–64. 10.1016/j.jinorgbio.2016.02.022 27031494 PMC12384776

[33] Davis S, Weiss M, Wong J, Lampidis T, Chen L. Mitochondrial and plasma-membrane potentials cause unusual accumulation and retention of rhodamine-123 by human breast adenocarcinoma-derived MCF-7 cells. J Biol Chem. 1985;260:13844–50. 4055760

[34] Sivapackiam J, Harpstrite SE, Prior JL, Gu H, Rath NP, Sharma V. Synthesis, molecular structure, and validation of metalloprobes for assessment of MDR1 P-glycoprotein-mediated functional transport. Dalton Trans. 2010;39:5842–50. 10.1039/c002361b . 20505882

[35] Piwnica-Worms D, Kronauge JF, Delmon L, Holman BL, Marsh JD, Jones AG. Effect of metabolic inhibition on technetium-99m-MIBI kinetics in cultured chick myocardial cells. J Nucl Med. 1990;31:464–72. 2324822

[36] Jayaraman P, Sada-Ovalle I, Nishimura T, Anderson AC, Kuchroo VK, Remold HG, et al IL-1beta promotes antimicrobial immunity in macrophages by regulating TNFR signaling and caspase-3 activation. J Immunol. 2013;190(8):4196–204. Epub 2013/03/15. 10.4049/jimmunol.1202688 23487424 PMC3622150

[37] Minotti G, Menna P, Salvatorelli E, Cairo G, Gianni L. Anthracyclines: molecular advances and pharmacologic developments in antitumor activity and cardiotoxicity. Pharmacol Rev. 2004;56(2):185–229. Epub 2004/06/01. 10.1124/pr.56.2.6 . 15169927

[38] Capranico G, Tinelli S, Austin CA, Fisher ML, Zunino F. Different patterns of gene expression of topoisomerase II isoforms in differentiated tissues during murine development. Biochim Biophys Acta. 1992;1132(1):43–8. Epub 1992/08/17. . 1380833 10.1016/0167-4781(92)90050-a

[39] Khiati S, Dalla Rosa I, Sourbier C, Ma X, Rao VA, Neckers LM, et al Mitochondrial topoisomerase I (top1mt) is a novel limiting factor of doxorubicin cardiotoxicity. Clin Cancer Res. 2014;20(18):4873–81. Epub 2014/04/10. 10.1158/1078-0432.CCR-13-3373 24714774 PMC4167185

[40] Zhang S, Liu X, Bawa-Khalfe T, Lu LS, Lyu YL, Liu LF, et al Identification of the molecular basis of doxorubicin-induced cardiotoxicity. Nat Med. 2012;18(11):1639–42. Epub 2012/10/30. 10.1038/nm.2919 . 23104132

[41] Kim SY, Kim SJ, Kim BJ, Rah SY, Chung SM, Im MJ, et al Doxorubicin-induced reactive oxygen species generation and intracellular Ca2+ increase are reciprocally modulated in rat cardiomyocytes. Exp Mol Med. 2006;38(5):535–45. Epub 2006/11/03. 10.1038/emm.2006.63 . 17079870

[42] Raj S, Franco VI, Lipshultz SE. Anthracycline-induced cardiotoxicity: a review of pathophysiology, diagnosis, and treatment. Current treatment options in cardiovascular medicine. 2014;16(6):315 Epub 2014/04/22. 10.1007/s11936-014-0315-4 . 24748018

[43] Ghigo A, Li M, Hirsch E. New signal transduction paradigms in anthracycline-induced cardiotoxicity. Biochim Biophys Acta. 2016;1863(7 Pt B):1916–25. Epub 2016/02/02. 10.1016/j.bbamcr.2016.01.021 . 26828775

[44] Kuznetsov AV, Margreiter R, Amberger A, Saks V, Grimm M. Changes in mitochondrial redox state, membrane potential and calcium precede mitochondrial dysfunction in doxorubicin-induced cell death. Biochim Biophys Acta. 2011;1813(6):1144–52. Epub 2011/03/17. 10.1016/j.bbamcr.2011.03.002 . 21406203

[45] Yang L, Luo C, Chen C, Wang X, Shi W, Liu J. All-trans retinoic acid protects against doxorubicin-induced cardiotoxicity by activating the ERK2 signalling pathway. Br J Pharmacol. 2016;173(2):357–71. Epub 2015/10/29. 10.1111/bph.13377 26507774 PMC5341234

[46] Takayama S, Ostuni E, LeDuc P, Naruse K, Ingber DE, Whitesides GM. Subcellular positioning of small molecules. Nature. 2001;411(6841):1016 10.1038/35082637 . 11429594

[47] Vilas GL, Corvi MM, Plummer GJ, Seime AM, Lambkin GR, Berthiaume LG. Posttranslational myristoylation of caspase-activated p21-activated protein kinase 2 (PAK2) potentiates late apoptotic events. Proc Natl Acad Sci U S A. 2006;103(17):6542–7. 10.1073/pnas.0600824103 16617111 PMC1458920

[48] Shrivastava A, Tiwari M, Sinha RA, Kumar A, Balapure AK, Bajpai VK, et al Molecular iodine induces caspase-independent apoptosis in human breast carcinoma cells involving the mitochondria-mediated pathway. J Biol Chem. 2006;281(28):19762–71. 10.1074/jbc.M600746200 . 16679319

[49] Giantris A, Abdurrahman L, Hinkle A, Asselin B, Lipshultz SE. Anthracycline-induced cardiotoxicity in children and young adults. Crit Rev Oncol Hematol. 1998;27(1):53–68. . 9548017 10.1016/s1040-8428(97)10007-5

[50] Doroshow JH. Effect of anthracycline antibiotics on oxygen radical formation in rat heart. Cancer Res. 1983;43(2):460–72. . 6293697

[51] Di Lisa F, Ziegler M. Pathophysiological relevance of mitochondria in NAD(+) metabolism. FEBS Lett. 2001;492(1–2):4–8. . 11248227 10.1016/s0014-5793(01)02198-6

[52] Ichas F, Jouaville LS, Mazat JP. Mitochondria are excitable organelles capable of generating and conveying electrical and calcium signals. Cell. 1997;89(7):1145–53. . 9215636 10.1016/s0092-8674(00)80301-3

[53] Bernardi P, Krauskopf A, Basso E, Petronilli V, Blachly-Dyson E, Di Lisa F, et al The mitochondrial permeability transition from in vitro artifact to disease target. The FEBS journal. 2006;273(10):2077–99. 10.1111/j.1742-4658.2006.05213.x . 16649987

[54] Mughal W, Dhingra R, Kirshenbaum LA. Striking a balance: autophagy, apoptosis, and necrosis in a normal and failing heart. Current hypertension reports. 2012;14(6):540–7. Epub 2012/09/25. 10.1007/s11906-012-0304-5 . 23001875

[55] Ichikawa Y, Ghanefar M, Bayeva M, Wu R, Khechaduri A, Naga Prasad SV, et al Cardiotoxicity of doxorubicin is mediated through mitochondrial iron accumulation. J Clin Invest. 2014;124(2):617–30. Epub 2014/01/03. 10.1172/JCI72931 24382354 PMC3904631

[56] Kauffman ME, Kauffman MK, Traore K, Zhu H, Trush MA, Jia Z, et al MitoSOX-Based Flow Cytometry for Detecting Mitochondrial ROS. React Oxyg Species (Apex). 2016;2(5):361–70. 10.20455/ros.2016.865 PubMed Central PMCID: PMCPMC5926237. 29721549 PMC5926237

[57] Polster BM, Nicholls DG, Ge SX, Roelofs BA. Use of potentiometric fluorophores in the measurement of mitochondrial reactive oxygen species. Methods Enzymol. 2014;547:225–50. 10.1016/B978-0-12-801415-8.00013-8 25416361 PMC4484872

