# Supplementary figures and images for: 68Ga-Galmydar: A PET imaging tracer for noninvasive detection of Doxorubicin-induced cardiotoxicity
Source: PLoS One. 2019 May 23;14(5):e0215579. doi: 10.1371/journal.pone.0215579 (PMC6532866; doi:10.1371/journal.pone.0215579)

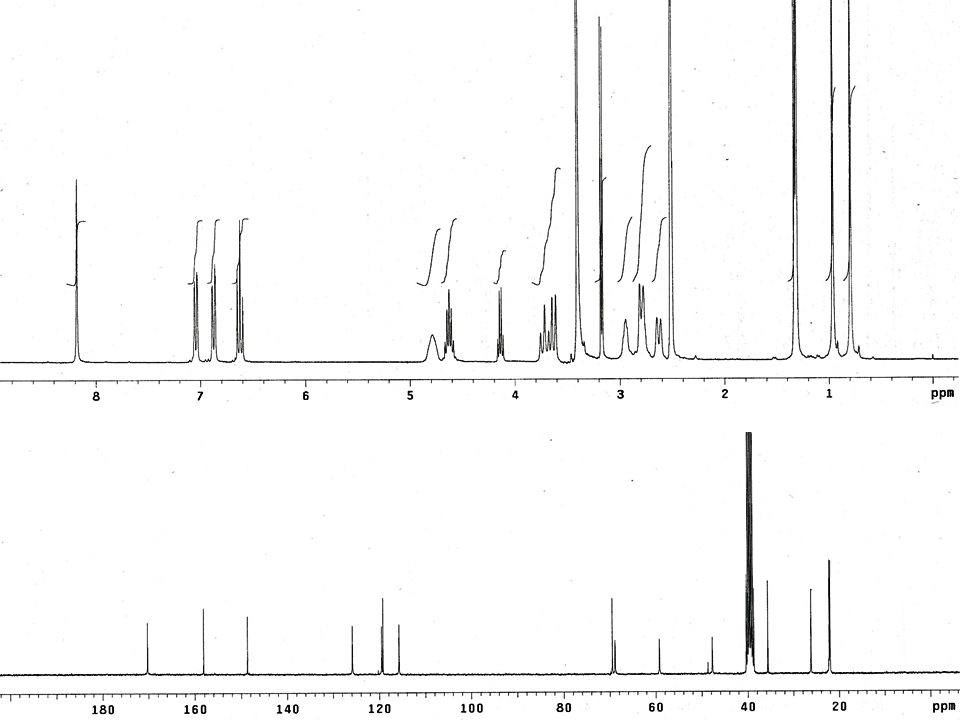

Supplement: S1 Fig — NMR Spectra of Galmydar in DMSO-d6: 1H NMR (Top); 13C NMR (Bottom). (TIF) [file pone.0215579.s001.tif]

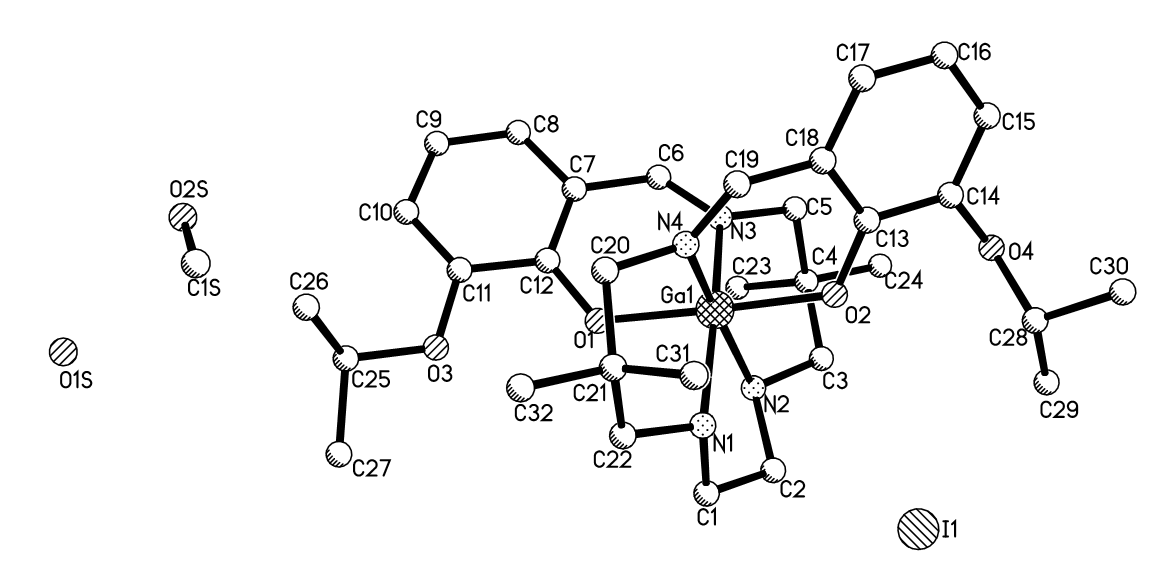

Supplement: S2 Fig — (TIF) [file pone.0215579.s002.tif]

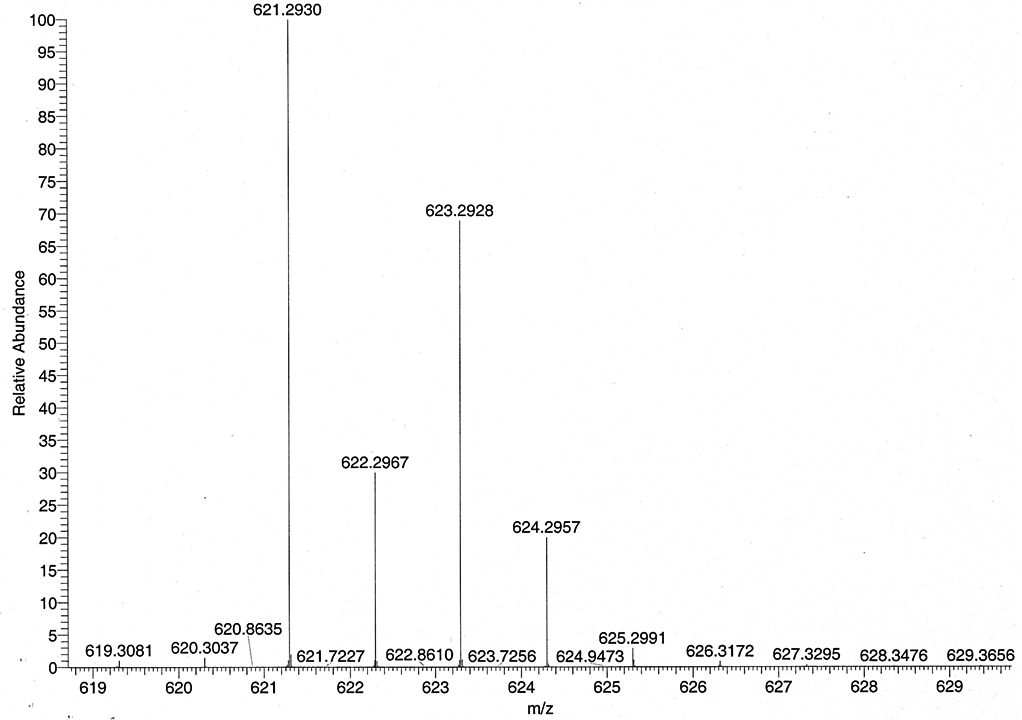

Supplement: S3 Fig — (TIF) [file pone.0215579.s003.tif]
