# Supplementary material for: 68Ga-Galmydar: A PET imaging tracer for noninvasive detection of Doxorubicin-induced cardiotoxicity
Source: PLoS One. 2019 May 23;14(5):e0215579. doi: 10.1371/journal.pone.0215579 (PMC6532866; doi:10.1371/journal.pone.0215579)
Supplement: S1 Table — (DOCX) [file pone.0215579.s004.docx]

**S1 Table**

| **Element** | **Atomic %**  **(Result)** | **Method** |
| --- | --- | --- |
| C | 50.51 | GLI Procedure ME-12 |
| H | 6.68 | GLI Procedure ME-12 |
| Ga | 9.05 | GLI Procedure ME-70 |
| N | 7.08 | GLI Procedure ME-12 |
