# Supplementary material for: 68Ga-Galmydar: A PET imaging tracer for noninvasive detection of Doxorubicin-induced cardiotoxicity
Source: PLoS One. 2019 May 23;14(5):e0215579. doi: 10.1371/journal.pone.0215579 (PMC6532866; doi:10.1371/journal.pone.0215579)
Supplement: S2 Table — (DOCX) [file pone.0215579.s005.docx]

**S2 Table** Theoretical calculations of elements and observed values (%) with errors calculated for analyzed elements.

| **Element** | **Galmydar** | **CH_3_OH** | **Total** | **M.Wt** | **Theoretical (%)** | **Observed (%)** | **Error (%)** |
| --- | --- | --- | --- | --- | --- | --- | --- |
| **C** | 32 | 1 | 33 | 396.363 | 50.72296 | **50.51** | 0.212961 |
| **H** | 48 | 4 | 52 | 52.41288 | 6.707327 | **6.68** | 0.027327 |
| **Ga** | 1 |  | 1 | 69.723 | 8.92252 | **9.05** | -0.12748 |
| **I** | 1 |  | 1 | 126.9045 | 16.24009 |  |  |
| **N** | 4 |  | 4 | 56.0268 | 7.169804 | **7.08** | 0.089804 |
| **O** | 4 | 1 | 5 | 79.997 | 10.23729 |  |  |
